# On the theory of mental representation block. a novel perspective on learning and behavior

**DOI:** 10.1080/19420889.2021.1898752

**Published:** 2021-03-12

**Authors:** Tobore Onojighofia Tobore

**Affiliations:** Independent Scholar

**Keywords:** Learning, behavior, evolution, memory, consolidation, reconsolidation, extinction, energy Efficiency, brain’s Resistance to Change, schema, mental Representation Block, why people believe in God, teleological argument; Cognitive Biases, creativity, psychiatric, cognitive and Neurodegenerative Diseases, sleep, synaptic Transmission, physician misdiagnosis, racism, homophobia, transphobia, indoctrination, deception, conditioning, close-mindedness, love, hate, artificial Intelligence, neural network algorithm

## Abstract

Understanding the mechanisms behind memory, learning, and behavior is crucial to human development and significant research has been done in this area. Classical and operant conditioning and other theories of learning have elucidated different mechanisms of learning and how it modulates behavior. Even with advances in this area, questions remain on how to unlearn faulty ideas or extinguish maladaptive behaviors. In this paper, a novel theory to improve our understanding of this area is proposed. The theory proposes that as a consequence of the brain’s energy efficiency evolutionary adaptations, all learning following memory consolidation, reconsolidation, and repeated reinforcements or strengthening over time, results in a phenomenon called mental representation block. The implications of this block on learning and behavior are significant and broad and include cognitive biases, belief in a creator or God, close-mindedness, dogmatism, physician misdiagnosis, racism, homophobia, and transphobia, susceptibility to deception and indoctrination, hate and love, artificial intelligence and creativity.

## Introduction

Learning is the process by which new understanding, behaviors, attitudes, knowledge, and ideas are acquired via unconscious and conscious pathways [1]. It has also been described as a relatively permanent modification in behavior achieved from practice or experience [2] and as the process by which a relatively stable change in stimulus-response relations is achieved as a product of environmental interaction via the senses [2].

Several theories of learning have been proposed including classical and operant conditioning, social learning theory, and cognitive theory. Classical conditioning which is associated with the Russian physiologist, Ivan Pavlov, is the process in which a neutral or unconditional stimulus becomes a conditioned stimulus after being connected or associated with an unconditioned stimulus [3]. Operant conditioning was made popular by B. F. Skinner in 1937 and he described it as behavior “controlled by its consequences”. Operant conditioning is the study of reversible behavior preserved by timed reinforcements [4]. Sociocultural or social learning theory describes learning to be a social process where learning occurs in a social context i.e. between people and their environment [5]. Cognitive theory focuses on internal cognitive changes due to the acquisition of knowledge [6]. It describes learning as involving the use of cognitive tools, such as insight, perceptions, information processing, and memory, to promote learning by assigning meaning to events [5,6].

Although, advances have been made on how our understanding of learning and behavior, questions remain on the difficulties in unlearning faulty ideas or extinguishing maladaptive behaviors. Annually, significant sums of money are spent on programs designed to facilitate the unlearning of maladaptive behaviors with poor results [7]. The big question, therefore, is why are learned behaviors so difficult to unlearn or extinguish? Unlearning faulty ideas or successfully extinguishing maladaptive behavior is crucial for individual successful development. It is also important for organizational and national development. The need, therefore, to better understand learning, memory, and behavior change cannot be emphasized.

The objective of this paper is to propose a novel theory of learning by describing how the processes of evolution and memory consolidation modulates behavior such that learned behavior becomes difficult to unlearn.

## Memory Formation, Consolidation, and Resilience to Disruption

The formation, preservation, and use of memories are crucial for normal functioning in the environment. Indeed, memory is essential for many purposes including decision-making, thinking, the fulfillment of goal-directed behavior, and problem-solving. Most importantly, it significantly contributes to the molding of human character and personality [8].

Memory formation begins with learning. At first, learned memories are subject to interference or labile, but become stabilized over time and firm at a cellular level such that they become resilient or resistant to disruption [9,10] through a process called memory consolidation [9–12]. Consolidation involves both molecular and cellular processes that modulate synaptic efficacy coupled with crosstalk between the hippocampus and cortex [12]. Furthermore, memory consolidation involves the crosstalk between the amygdala, prefrontal cortex, and the medial temporal lobe in an integrated manner [13]. Emotion has a significant modulatory effect on learning and memory and can enhance or disrupt learning and memory consolidation [13,14].

Memory can go also through a process of reconsolidation [15]. Reconsolidation entails the returning of an established memory to a transiently labile state for modification and strengthening or weakening after which is it stabilized or consolidated again rendering it resilient to disruptions [8–10,16]. Reconsolidation has been described as not fundamentally distinct from consolidation but as a phase in memory consolidation process [17]. The strength and age of the memory play a critical role in the stability of memory and its resilience to disruption and reconsolidation, suggesting a temporal gradient of progressive resistance to memory disruption post reactivation. Indeed, weak and young memories are vulnerable to post-reactivation disruption, in contrast, older memories are more resilient to disruption [9,18–22].

Extinction is one of the ways to change learned behavior [23,24]. However, multiple lines of evidence indicate that extinguished behavior returns under many conditions [23,24], reinforcing the resilience of well-consolidated memory. Evidence suggests that memories are at first dependent on the hippocampus and distributed regions of neocortex but as it gradually goes through the process of consolidation, connections among the cortical regions are steadily reinforced until the cortical memory is autonomous of the hippocampus [25–27]. Importantly, damage to the hippocampal region causes a temporally limited retrograde amnesia and damage to medial temporal lobe structures causes significant retrograde amnesia covering decades, reinforcing the temporal gradient of memory consolidation [28].

## Energy Efficiency Evolutionary Adaptation of Well Consolidated Memory

Learning and memory consolidation are energy-expensive processes [29]. Indeed, consolidation and reconsolidation require de novo protein synthesis [15,30–33] and protein synthesis is highly energy-expensive [34]. Learning and memory involve a cascade of molecular energy-expensive events in dendritic spines and to be stored, longer memories typically require proportionally more energy [35]. Furthermore, memory formation involves synaptic plasticity or the alteration in the transmission efficacy at the synapse [36,37]. Notably, of the brain’s many components, synapses and synaptic neurotransmission are the most energy-expensive [38,39], and neurotransmitters including monoamines, GABA, glutamate, and acetylcholine are critical in learning and memory consolidation [40–45]. Astrocytes have been shown to play a critical role in learning, cognition, and memory consolidation [46–50], and evidence suggests that they bear the brunt of the brain’s metabolic load [51]. Sleep plays a crucial role in memory consolidation [52–54], and evidence suggests that sleep is an energy-expensive process [55]. Indeed, rapid eye movement (REM) plays a critical role in memory consolidation and research suggests that it is as energy-expensive as wakefulness [55,56]. In Drosophila flies, upregulation, or elevation of energy intake in neurons in the fly’s major memory center is critical to the consolidation of long-term memory [57].

Memory formation and consolidation are metabolically expensive processes, and this increases the need for energy efficiency evolutionary adaptations. Indeed, the availability of energy imposes a significant evolutionary constraint on the brain’s capabilities [58–61]. Evidence suggests that energy efficiency is one of the critical factors that guide the evolution of species and the brain and nervous systems have evolved adaptations to be highly energy-efficient [59–65]. Importantly, research indicates that the increased resilience or resistance to disruption of memory following consolidation and reconsolidation is an evolutionary adaptation of the brain due to energy efficiency [29]. Although the brain represents only 2% of the total body mass, approximately 20% of the oxygen and a quarter of the total glucose consumed by the human body are dedicated to brain functions [66]. Without this increased resilience to memory disruption following reconsolidation and consolidation, the brain is likely to use up much more of the body’s energy as it must constantly relearn memory crucial to the organism’s survival because of their easy or persistent disruption [29]. This would be hugely energy expensive and could hamper the brain’s ability to handle other critical functions necessary for the survival of the organism [29].

## Memory Resilience to Disruption, Memory Network and Mental Representation Block (MRB)

New memories are not acquired or learned in isolation but interleaved within a huge network of relevant preexisting knowledge or schema [10,67]. Indeed, initial consolidation occurs via a reordering of preexisting memories, and schemas involve the interleaving of new learning with preexisting memories and later with future memories [10]. Strong evidence suggests that schema development and streamlining play an essential role in memory consolidation [67] and schemas do not differentiate episodic and semantic memories, but simply interleave all memories through common elements [10]. Also, and importantly, as information or knowledge goes through the memory consolidation and reconsolidation process, it gains in familiarity, trustworthiness, and value. In other words, the network of interleaved memories could be called the “network of the familiar and the trusted”

The major implication of the brain’s energy efficiency evolutionary adaptation that makes well-consolidated memory resilient to disruption is the creation of a mental depiction or representation block (MRB). MRB is an emergent phenomenon of the brain due to the energy efficiency evolutionary adaptation of well-consolidated memory’s resilience or resistance to disruption that causes the brain to justifiably and inflexibly believe in the correctness of an idea or information within the limits of the way it is depicted or represented in memory and the interleaved network of memories and to resist contrary ideas or information. The brain’s network of interleaved memories or schema contributes to an individual’s identity, personality, and character, and it is instrumental in shaping their worldview [8]. It is made up of memories that have been reinforced, consolidated, and reconsolidated for decades, and this required significant energy expenditure. The familiarity and trustworthiness of well-consolidated memories contribute to the MRB, as the need to expend energy by disrupting and downgrading well-consolidated memories and consolidate and elevate the unfamiliar or distrusted is resisted or avoided. So, the brain makes every effort to maintain the status quo and that effort is more potent for salient memories or memories which are strongly consolidated and associated with many other memories in the network.

Indeed, research indicates that information or knowledge that is congruent or associated with preexisting knowledge or schema is consolidated quickly [68,69], and familiarity, trustworthiness, and energy efficiency [29], are the reason for the tendency to quickly consolidate information that is congruent with preexisting knowledge or the network of interleaved memory. Similarly, prior knowledge can significantly enhance memory processes involved in acquiring novel knowledge but can also impede knowledge acquisition, particularly when the information to be acquired or learned is not consistent with the learner’s presuppositions [70].

The implications of MRB are significant and broad. The most important implication is that all learning including associative learning, operant, and social conditioning, etc. following memory consolidation, reconsolidation, and repeated reinforcements over time, results in MRB. MRB causes inflexibility observed in knowledge acquisition and this is more pronounced when the person is an expert or sophisticated on the subject. This entails that the stronger and/or older the consolidated memory, the more trustworthy it is, and the more energy-expensive it is to disrupt or change it, and the greater the resistance to change. Indeed, extreme beliefs elicit potent dogmatic intolerance [71]. Evidence suggests that perceptions of expertise promote closed-minded cognition and cause people to be unwilling to support contrary points of view and to think inflexibly [72]. Similarly, strong evidence indicates that prior attitude plays a critical role in people’s attitudes and beliefs such that attitudinally incongruent arguments are judged as inferior to attitudinally congruent arguments leading to confirmation and disconfirmation biases and attitude polarization [73]. Notably, this attitude polarization was found to be more powerful among those with the strongest prior knowledge and highest levels of political sophistication [73], reinforcing the strong relationship between in-depth understanding or expertise and the tendency to be less willing to embrace or engage counterarguments.

MRB can be used to understand certain emotions and behavior associated with love and hate. Love is associated with emotional dependence, aggrandizement, or magnification of a partner’s traits and worth, trivializing of his or her faults, and obsessive thinking [74,75]. Hate, on the other hand, is associated with the inability to see good in the person or object of hate, repulsion, disgust, distancing, and a lack of concern or compassion for the welfare or wellbeing of the person [76]. Evidence suggests that emotion has a profound modulatory effect on learning and memory [13,14], and love and hate are associated with strong emotional memories such as trust, mistrust, fear, repulsion, disgust, and anger. The representation of the love or hate object in memory is extraordinarily strong and this creates a potent MRB that restricts the individual to in many cases unobjectively or irrationally associate the hate object with all things negative and the love object with all things positive. The evolutionary benefit for this is to protect the individual from harm.

Moreover, MRB makes the brain to be highly susceptible to deception and trickery. A person is likely to fall victim to deception and even resist all warnings not to proceed or engage a fraudster if the fraudster has already earned their trust or looks and acts in ways the victim understands in memory to be trustworthy or credible. MRB also makes the brain susceptible to indoctrination, propaganda and brainwashing. Information control and relentless negative, or positive portrayal can significantly reinforce how the world works in memory such that it affects behavior and could cause people to resist contrary information. Indeed, evidence suggests a direct role of hate propaganda in incitement to cause harm [77,78]. The length of exposure, pervasiveness, or saturation of the information and the sophistication of a person on the subject modulates the brain’s vulnerability to propaganda and deception. Repeated exposure to multiple lines of evidence that reinforce the trustworthiness of the idea or information, being in an environment that continuously reinforces the trustworthiness of the idea, prevention from accessing and trusting contrary information, and application of positive and negative reinforcements can significantly strengthen the MRB.

Furthermore, MRB could be used to understand why people believe in God or an intelligent supernatural being even without religious and social conditioning. Teleological arguments for the existence of a supernatural being or god are based on the complexity, exquisiteness of function, structure, and interconnectedness in the natural world and this makes many people see a deliberative and directive supernatural mind behind these complexities, order, and beauty [79–81]. Design, beauty, order, and complexity are strongly associated with the work of a designer or intelligent being in our everyday lives and this understanding is strongly reinforced and well consolidated in memory. This creates a strong MRB that results in a tendency to see the order, complexity, and exquisiteness in nature as the work of a supernatural intelligent being and to both resist and see as illogical ideas that suggest that these events or phenomena happened by chance. In addition, MRB due to human experience of causality explains the appeal for the cosmological argument of a God.

## Discussion

It is well known that humans are resistant to change [82] but why this happens is not fully understood at the level of the brain and it has never been explained from the perspective of energy efficiency evolutionary adaptation of well-consolidated memory’s resilience to disruption. Although schema theory has been used to provide an appealing and descriptive framework for making sense of human knowledge processing and how it influences behavior, it remains an ill-constrained theoretical construct that provides scant detailed process assumptions [83,84].

In summary, as a consequence of the brain’s energy efficiency evolutionary adaptation, all learning whether from classical, operant or social conditioning, etc. following memory consolidation, reconsolidation, and repeated reinforcements over time, results in MRB and this makes the brain resistant to ideas that are contrary to prior knowledge or not in conformity with understood reality. MRB provides a novel way to understand why the brain is resistant to ideas that are contrary to prior knowledge or not in conformity with understood reality. It helps us understand at the level of the brain why people defend acquired faulty ideas, may be unwilling to engage with contrary information, and why they cannot simply give up such faulty ideas even in the face of evidence or persuasion. MRB makes the brain a biased‐information processor. Indeed, the brain could be thought of as forming a shield or umbrella of bias for incoming information and it is highly selective of the information that it commits to memory, interleaves within the huge network of relevant preexisting knowledge or schema, and elevates as part of the individual’s belief system. This has a profound effect on learning and behavior. It could be used to understand many human behaviors including different forms of biases, functional fixedness, physician misdiagnosis, racism, transphobia, and homophobia, and ways to overcome it could serve as the basis or foundation for learning and new teaching methods.

MRB allows the brain to use its energy resources more efficiently and to protect familiar and trustworthy information critical to the organism’s survival in its environment. Consequently, alterations in the energy efficiency of memory consolidation, reconsolidation, and extinction process may be the basis of certain diseases or conditions associated with the brain. Indeed, neurodegenerative diseases, cognitive and psychiatric conditions, including unipolar and bipolar depression, anxiety, schizophrenia, and posttraumatic stress disorder are associated with alterations in memory consolidation and extinction. Alteration in factors critical in memory consolidation including adult neurogenesis, sleep, redox homeostasis [85], and neurotransmitters activity are implicated in memory deficits, psychiatric and cognitive disorders, and neurodegenerative diseases, indicating a deep connection between all of them.

Also, memory is involved in creativity [86]. Indeed, free-associative episodic thinking is intertwined with creative operations [87] and imagination and creativity play a role in allowing the brain to think beyond its repertoire of knowledge and understanding. However, it is unclear how creativity and imagination may interact to override restrictions caused by MRB in decision-making and behavior. Perhaps, it is because the creative brain is not very energy efficient. Indeed, the developing brain of a child is not as energy efficient as an adult brain and it is believed to be more creative [88]. This might cause the creative brain to have a higher metabolic load and evidence suggests that the metabolic requirements of the developing human brain are about half of the total body’s daily energy requirement [89,90]. If the creative brain is more able to override MRB it could also be more likely to be rebellious against convention and traditionalism. Importantly, creativity is linked to dishonesty, and dishonesty could lead to greater creativity [91,92]. In addition, the energy inefficiency and increased metabolic load of the creative brain could potentially increase the likelihood of mood or mental health problems. Notably, creativity is linked to psychopathology [93,94] and the developing human brain is highly vulnerable to mental illness [95,96].

MRB has implications for artificial intelligence (AI). AIs and large neural networks require monumental computational resources that correspondingly necessitate substantial energy consumption [97,98], indicating that energy efficiency is a major problem in the advancement of AI technology. In recent years, there have been discussions about AI and neural network algorithms with similarities to human cognition [99] posing a threat to humanity. Such a machine will require access to unlimited energy resources and possess highly efficient neural networks than the human brain. In addition, it must be able to quickly overcome MRB by being able to rapidly generate new ideas in the face of challenges significantly faster than the human brain, downgrade trusted information it has committed to memory more rapidly than the human brain, and permanently keep an open mind. Building such a machine is unfeasible with the currently available technology and perhaps impossible in the future.

To overcome the restrictions created by an MRB, it is crucial to change the individual’s environment if possible and most importantly, create conditions or expose them to irrefutable evidence from multiple lines that will cause them to hold with less regard and trust, the idea, information, person or issue in question. Success is dependent on the level of sophistication or expertise of the person. In other words, success is likely to be gradual especially for older and strongly consolidated ideas entrenched in memory that make up the core part of a person’s beliefs because of the high trustworthiness and energy expenditure associated with them. To prevent MRB due to faulty ideas and information, it is important to teach people all sides of an issue and clearly explain the strengths and weaknesses of each option to stimulate critical thinking. To avoid MRB created by false knowledge or information, better outreach and teaching methods to people particularly children and young adults whose ideas of the world around them are still formative is crucial to individual, organizational and national development. Better and more innovative teaching methods are needed that are not too focused on rote memorization and learning. Traveling overseas should be mandatory as part of the school curriculum to expose young people to different cultures and values and broaden their understanding. Indeed, travel is associated with broadening the mind and enhancing creativity [100–103].

In conclusion, it is important to note that memory is dynamic and the age and strength of memory are not insurmountable barriers to memory reconsolidation [104]. Indeed, if the reactivation session is extensive or prolonged, well-consolidated memory could be disrupted [9], indicating that although old and strong memory may be resilient to disruption, under certain conditions they could face extinction and reconsolidation. Also, there is a spectrum to the potency of MRB. This spectrum is modulated by the length, depth, and frequency of the reinforcement of the learned information, stability/variability of the conditions (favorable/adverse) of the individual and the environment in regard to their wellbeing and the length, depth, and frequency of exposure to the contrary or alternative. In a controlled environment where the conditions of the individual and environment are stable and favorable, it is easier to achieve and maintain MRB. There are several limitations to the theory. Firstly, questions remain about the role of protein synthesis in memory consolidation [105]. Moreover, people avoid engaging information that is incongruent to their beliefs for many reasons including the need to maintain relationships with friends or colleagues with different opinions [106] and in pursuit of a preferred conclusion [107]. The evidence linking creativity with mental illness remains inconclusive [108] and there are disagreements about the temporal constraints on memory reconsolidation [15,109,110]. It is unclear at which point MRB is formed following memory consolidation and repeated reinforcements. In addition, it is unclear the exact amount and length of exposure to contrary information as well as the type of personal experience needed to overcome an MRB. Finally, it is possible that there are individual variations in the formation of MRB and this could be an area of interest for future research.

## A Simple Experiment to test MRB

There are several possible ways to test MRB. The goal is to condition the animal(s) or person(s) to learn about an idea or information for some time until it is well consolidated in memory and then introduce a contrary idea or information. An example would be a large simple trial of people in a hotel or hostel complex. Subjects should not be allowed to speak to each other and should be of similar age and educational level. They should be randomly selected to stay in different rooms. Each room should have a standard bedroom and bathroom, and, in the bathroom, there should be either an automatic paper dispenser with an inconspicuous nonworking button at the side or both the automatic paper dispenser and a similar-looking manual paper dispenser that works by pressing the inconspicuous side button that is nonfunctional in the automatic paper dispenser. Subjects already exposed to a paper dispenser that resembles or works exactly like the manual paper dispenser prior to the study should be excluded from the study. A questionnaire should be filled daily by all subjects detailing their sleep, experience using the paper dispenser, and general well-being.

Subjects should be individually introduced to using the paper dispenser(s) in their rooms as part of their introductory welcome and should be instructed to use the paper dispenser(s) daily. After a few weeks (ideally a month or longer), the automatic paper dispensers should be removed only from rooms that have both types of paper dispensers and all subjects should be randomly reassigned to different rooms. Most or all subjects exposed for the first time to the manual paper dispenser that works by pressing the inconspicuous side button that was nonfunctional in the automatic paper dispenser should complain that the paper dispenser in their new room is faulty or out of paper. Even when repeatedly told that the paper dispenser is in good working condition, they will conclude that it is faulty or out of paper. Subjects exposed to using both types of dispensers should have no problem using the paper dispenser in their new rooms.
